# Memorial of the National Medical Society of the District of Columbia

**Published:** 1870-02

**Authors:** 


### Memorial of the National Medical Society of the District of Columbia.

*To the Members of the Senate and House of Representatives of the United States:*

Whereas it has been stated in a published circular that the persons endeavoring to from a medical society on the basis of " equality before the law" have maliciously and falsely attacked the Medical Society of the District of Columbia, we deem it but just to the public, as well as ourselves, to make the following statement of facts :

Within the past few years some colored physicians regular graduates of medical colleges. and of untarnished character and reputation, having held positions as surgeons in the Union army during the rebellion, have settled in this city and secured to themselves a large professional practice.

There being only one medical society in the District where all licenses to practice must be obtained, and all advantages flowing from medical and professional discussions were to be enjoyed, it became the duty of these colored physicians to obtain license and membership, in order to keep up their medical education, and derive all the advantages from weekly professional discussions.

The Medical Society of the District of Columbia has, on two different occasions, refused to elect these colored physicians to membership, acknowledging that the color of the candidates was the reason for so doing, and some of its members have refused to consult with them because they were not members of the Society.

This was in June, 1869. Hoping that discussion of the subject would aid in securing justice. we were content to await the result.

*Junuary* 3, 1870, by a vote of 26 to 10, the Society refused to consider a resolution offered by Dr. Reyburn, which read as follows, viz :

" *Resolved*, That no physician (who is otherwise eligible) should be excluded from membership in this society on account of his race or color."

Some of the present officers of the Society have refused to consult with the colored physicians, but instead thereof, have taken charge of patients who were under their care, without giving them the customary notice of dismissal, in direct violation of the ethics of the profession.

These colored physicians have applied to the Society for membership, but were rejected, by a large majority, although the Board of Examiners reported favorably on them. At the last election of officers in the Society, *held January* 3, 1870, *the Chairman of this Board* was removed, and a gentleman, late of the Confederate army, well known for his opposition to the admission of colored physicians, was elected in his place, thus insuring their future defeat. Other gentlemen who served during the war in the Confederate army are now prominent in the control of its affairs.

At the same meeting a white candidate, a gentleman of high professional standing, and occupying an important official position, was objected to, solely on the ground that he was believed to be in favor of the admission of colored members.

Again, the circular published by the committee of the Society, states that their weekly meetings are "social reunions." These meetings are conducted under strictly parliamentary rules, from the opening to the adjournment, and only professional questions, essays and papers, are brought forward for discussion, *and gentlemen are even required to obtain permission of the President to retire from the meeting.* If these meetings held in compliance with the charter of the Society, are only social reunions, then the meetings of all bodies not strictly parliamentary are social reunions.

Other colored men will soon graduate from medical colleges in the United States and throughout the world, and their rights should be protected and guaranteed within this District.

It is a fact worthy of note, that this is the only country and the only profession in which such a distinction is now made. Science knows no race, color or condition, and we protest against the Medical Society of the District of Columbia maintaining such a relic of barbarism.

We, for the reasons stated, and in accordance with the spirit of the times, ask Congress to grant a Charter to a new Society which will give all rights, privileges and immunities to all physicians, making only the presentation of a diploma from some college recognized by the American Medical Association, and good standing in the profession, the qualifications necessary for membership.

ROBERT REYBURN, *President.*

| | | |
|---|---|---|
| JOHN G. STEPHENSON, M. D., | C. B. PURVIS, M. D., | *Committee.* |
| ALEX. T. AUGUSTA, M. D. | R. J. SOUTHWORTH, M. D. | |
| D. W. BLISS, M. D., | JOS. T. JOHNSON, M. D. | |
| SILAS L. LOOMIS, M. D., | JOHN E. MASON, M. D. | |

C. ADAMS GRAY, M. D., *Secretary.*

